# Pitch discrimination is better for synthetic timbre than natural musical instrument timbres despite familiarity

**DOI:** 10.1121/10.0011918

**Published:** 2022-07-01

**Authors:** Emma Holmes, Elizabeth E. Kinghorn, Lucy M. McGarry, Elizabeth Busari, Timothy D. Griffiths, Ingrid S. Johnsrude

**Affiliations:** 1 Department of Speech Hearing and Phonetic Sciences, University College London, London WC1N 1PF, United Kingdom; 2 Don Wright Faculty of Music, University of Western Ontario, London, Ontario N6A 3K7, Canada; 3 Brain and Mind Institute, University of Western Ontario, London, Ontario N6A 5B7, Canada; 4 UCL Ear Institute, University College London, London WC1E 6BT, United Kingdom; 5 Biosciences Institute, Faculty of Medical Sciences, Newcastle University, Newcastle upon Tyne NE2 4HH, United Kingdom

## Abstract

Pitch discrimination is better for complex tones than pure tones, but how pitch discrimination differs between natural and artificial sounds is not fully understood. This study compared pitch discrimination thresholds for flat-spectrum harmonic complex tones with those for natural sounds played by musical instruments of three different timbres (violin, trumpet, and flute). To investigate whether natural familiarity with sounds of particular timbres affects pitch discrimination thresholds, this study recruited non-musicians and musicians who were trained on one of the three instruments. We found that flautists and trumpeters could discriminate smaller differences in pitch for artificial flat-spectrum tones, despite their unfamiliar timbre, than for sounds played by musical instruments, which are regularly heard in everyday life (particularly by musicians who play those instruments). Furthermore, thresholds were no better for the instrument a musician was trained to play than for other instruments, suggesting that even extensive experience listening to and producing sounds of particular timbres does not reliably improve pitch discrimination thresholds for those timbres. The results show that timbre familiarity provides minimal improvements to auditory acuity, and physical acoustics (e.g., the presence of equal-amplitude harmonics) determine pitch discrimination thresholds more than does experience with natural sounds and timbre-specific training.

## I. INTRODUCTION

The ability to discriminate small pitch differences may be particularly beneficial for musicians. For many orchestral instruments, a musician's ability to detect small differences in frequency is critical for playing the correct note and also, in some instances (e.g., violin), for tuning the instrument. The threshold, or “just noticeable difference” (JND), for discriminating pitch is often measured by presenting two sounds sequentially and asking listeners whether the two sounds have the same or a different pitch or which sound is higher or lower in pitch. Here, we asked whether natural experience with sounds of particular timbres improves pitch JNDs for sounds of those timbres over other, less familiar timbres.

The JND depends on acoustic properties of the sounds to be discriminated: Listeners can detect smaller differences in pitch between two harmonic complex tones than between two pure tones (Flanagan and Saslow, 1958; Micheyl *et al.*, 2006; Zeitlin, 1964), demonstrating that listeners utilize the harmonics of complex tones to improve pitch judgments. Better discrimination at particular frequencies cannot account for this finding, because the JND for a complex tone is better than the best pure-tone JNDs of its component frequencies (Faulkner, 1985). At the same time, pitch discrimination depends on the combination of harmonics that are present in a complex tone (Houtsma and Smurzynski, 1990; Miyazono and Moore, 2013; Moore *et al.*, 1992). Houtsma and Smurzynski (1990) presented complex tones with a missing fundamental of 200–300 Hz, containing a subset of harmonics (those above 7–25 in the harmonic series). JNDs were worse for complex tones that only contained frequencies above the 13th harmonic than for those that also contained lower frequencies (7–12 in the harmonic series). Thus, the timbre of a harmonic complex tone influences the smallest pitch differences that a listener can perceive.

Sounds in the natural world do not resemble the complex tones used in the aforementioned experiments, which have flat spectra or have had their harmonics selectively removed. Rather, the resonant properties of the production system (e.g., human vocal tract or body of a musical instrument) produce sounds with formants at the positions of resonances and, more generally, different intensities at different frequencies. On one hand, pitch discrimination could be worse for natural sounds. Natural sounds contain fewer harmonics than flat-spectrum complex tones, and this could potentially affect pitch perception: Bernstein and Oxenham (2003) found robust pitch discrimination for complex tones that only contained the ninth and higher harmonics, so these higher harmonics might aid pitch perception for sounds containing lower harmonics (although there is some evidence the higher harmonics contribute little to pitch perception among musicians: Dai, 2000; Moore *et al.*, 1985). Second, unequal intensities for the harmonics that are present (e.g., across the first five harmonics, which are thought to be most important for pitch perception: Dai, 2000; Moore *et al.*, 1985) may make it more difficult to extract pitch information from those harmonics. Other factors that could lead to worse pitch discrimination for natural sounds include greater variability (jitter) of pitch information, noisier information (i.e., less favorable harmonic-to-noise ratio), and the shape of the amplitude envelope. On the other hand, the ability to discriminate differences in pitch can be improved through training (Amitay *et al.*, 2005; Ari-Even Roth *et al.*, 2003; Atienza *et al.*, 2002; Delhommeau *et al.*, 2002; Demany, 1985; Demany and Semal, 2002; Grimault *et al.*, 2002; Irvine *et al.*, 2000; Menning *et al.*, 2000; Micheyl *et al.*, 2006; Sinnott *et al.*, 1985), demonstrating that discrimination thresholds are not fixed but are instead influenced by prior experience. Possibly, prior experience with sounds in the natural world might lead to better pitch discrimination for tones with natural (familiar) than unnatural (unfamiliar) spectra; this effect may be large enough to override the benefit obtained from the acoustic content of unnatural, flat-spectrum harmonic complex tones.

Based on previous studies that have measured pitch discrimination of sounds with natural spectra, it is unclear whether pitch discrimination is better or worse for natural than for artificial complex tones. Two studies measured pitch discrimination of artificial vowels (Flanagan and Saslow, 1958) and of a female speaking the vowel “ah” (Moore *et al.*, 2008). Flanagan and Saslow (1958) found better discrimination of artificial vowels than of pure tones, showing that the advantage for discriminating complex, compared to pure, tones also applies to sounds with more natural spectra. However, they did not compare discrimination of complex tones that had more artificial compared to more natural spectra. To our knowledge, Moore *et al.* (2008) was the only group to examine pitch discrimination using both natural (vocal) and artificial complex tones. They found no difference in pitch discrimination between the natural female voice and the artificial complex tone, although the vowel that was spoken by the female talker was missing word or sentence context that we naturally encounter, which may help us to discriminate pitch when listening to speech in natural settings. Other studies using natural [Pitt (1994), experiment 1; Vurma *et al.* (2011)] and artificial (Krumhansl and Iverson, 1992; Singh and Hirsh, 1992; Warrier and Zatorre, 2002) timbres have demonstrated that a *difference* in timbre between a pair of tones influences judgments of pitch. However, these papers do not statistically compare pitch discrimination between timbres for same-timbre pairs of tones [e.g., trumpet–trumpet pairs compared to piano–piano pairs in Pitt (1994)]. In a task in which participants classified pitch as high or low, Pitt (1994) (experiment 2) found no difference depending on whether the stimuli were trumpet or piano tones; however, the pitch difference (294 or 417 Hz) was large, and accuracy was >90% correct, so this null result might be explained by a ceiling effect.

Other studies have examined effects of timbre in pitch *interval* discrimination tasks (i.e., when participants are asked to discriminate the magnitude of the difference in pitch—in other words, the interval—between pairs of tones), although the findings are mixed. Zarate *et al.* (2013) compared pitch interval discrimination of pure tones, piano tones, flute tones, and synthetic voices. However, contrary to the aforementioned pitch discrimination tasks, they found that pitch interval discrimination was most accurate for pure tones. Russo and Thompson (2005) found that judgments of pitch interval size were affected by changes in artificial timbre between the first and second tone of the interval, but when the timbre was constant, pitch interval size judgments did not differ between “bright” and “dull” timbres.

The notes played by musical instruments vary naturally in timbre, and musicians have extensive experience with the musical instrument on which they are trained. Musical training might be considered a special instance, and more naturalistic form, of pitch discrimination training. Indeed, musicians perform substantially better (by a magnitude of 2–4) on pitch discrimination tasks than non-musicians, both when they are required to discriminate two tones that are presented sequentially (Besson *et al.*, 2007; Brown *et al.*, 2017; Kishon-Rabin *et al.*, 2001; Magne *et al.*, 2006; Micheyl *et al.*, 2006) and when they are required to discriminate tones in ten-tone sequences (Spiegel and Watson, 1984). Although musicians have not been found to discriminate timbre better than non-musicians (Allen and Oxenham, 2014), Micheyl *et al.* (2006) report a larger musician advantage for discriminating the pitches of complex compared to pure tones, consistent with the idea that musicians' natural experience with complex tones improves pitch discrimination most for complex tones and less for sounds that differ vastly in timbre from trained sounds (i.e., pure tones). Musicians might be expected to show better pitch discrimination for sounds of an instrument they have been trained to play, given that they have extensive experience making pitch judgments for the timbre of that instrument compared to other sounds they cannot produce. In other words, becoming highly familiar with sounds of a particular timbre may enable listeners to better extract pitch information specifically for sounds of that timbre compared to other, less familiar sounds (for example, due to better predictions about harmonic intensities). Consistent with this idea, Miyazaki (1989) found that piano players with absolute pitch were best at identifying the pitch of natural piano tones (91.6% correct), intermediate at identifying the pitch of artificial piano sounds (74.4%), and worst at identifying the pitch of pure tones (80.4%), although these results cannot distinguish whether this advantage reflects training on the piano specifically or a more general advantage for natural compared to artificial sounds. Also, effects of timbre on pitch identification in individuals with absolute pitch may differ from more general effects of timbre on pitch discrimination.

Several mechanisms could underlie better pitch discrimination of sounds played on instruments that musicians have been trained to play. Experience could improve pitch discrimination by enabling better predictions of where in the frequency spectrum or when in time (based on knowledge of the temporal envelope) to listen. Alternatively, musicians would be expected to have strong sensorimotor mappings for pitches of instruments that they play; these would be weaker or nonexistent for other instruments. Such mappings, which may provide another way to represent pitch information, might improve pitch discrimination. For instruments capable of fine changes in pitch (e.g., violin, flute, trumpet), these mappings may be even more fine-tuned for pitch; to play the correct pitch, musicians must make rapid motor (mouth or finger) micro-adjustments and be able to hear how these affect the pitch of the sound they produce. Indeed, Hafke-Dys *et al.* (2016) demonstrated that violinists are capable of compensating for perturbations in pitch while playing a series of notes, even when the pitch perturbations were lower than their perceptual thresholds measured when they heard but did not play the same notes. Musicians show enhanced neural responses to notes played on instruments that they play, compared to notes played on instruments they have never been trained to play (Pantev *et al.*, 2001; Shahin *et al.*, 2008; Strait *et al.*, 2012), which provides a neural substrate by which perception could be improved for trained instruments. Nevertheless, whether pitch discrimination is better for trained than untrained instruments is currently unknown.

Here, we compared musicians' and non-musicians' pitch discrimination thresholds for musical tones (played on the violin, trumpet, and flute) and synthetic flat-spectrum harmonic complex tones. We presented participants with two bars of a four-tone sequence that contained a deviant-pitch tone in the second bar. We showed participants musical notation for each four-tone sequence before the sequence began, so that they knew in advance the correct pitches of the tones. Consistent with previous findings (e.g., Besson *et al.*, 2007; Brown *et al.*, 2017; Kishon-Rabin *et al.*, 2001; Magne *et al.*, 2006; Micheyl *et al.*, 2006; Spiegel and Watson, 1984), we expected to find better pitch discrimination in musicians than non-musicians. Crucially, we recruited three groups of musicians who were trained to play one of the instruments (violin, trumpet, or flute) that they heard in the experiment, enabling us to compare differences in thresholds due to acoustics and familiarity. If thresholds are primarily determined by acoustic factors (e.g., spectral content, jitter, harmonic-to-noise ratio), pitch discrimination thresholds should be better for flat-spectrum harmonic complex tones than for musical tones, whereas, if the effect of experience is large enough to compensate for the spectral content of flat-spectrum harmonic complex tones, thresholds for musical tones should be as good or better than thresholds for complex tones—and be best for the musical instrument that the listener has learnt to play.

## II. METHODS

### A. Participants

We excluded data from four participants: three did not meet the criteria for normal hearing (average pure-tone thresholds averages greater than 20 dB hearing level (HL) at octave frequencies between 0.5 and 4 kHz), and one did not respond on 15% of trials. We analysed the data from 51 participants (16 male, 35 female) aged 18–58 years [median = 24.0 years, interquartile range (IQR) = 10.5]. All of these participants had average pure-tone thresholds at octave frequencies between 0.5 and 4 kHz of 15 dB HL or better in each ear. While nine participants had pure-tone thresholds at 4 or 8 kHz greater than 20 dB HL in at least one ear, these participants were spread across the groups (three non-musicians, four violinists, one trumpeter, and one flautist).

Non-musicians (*N* = 14) were required to have no more than 2 years of musical training: our non-musicians had either never been trained on an instrument (*N* = 9) or had learnt an instrument (the piano, recorder, or bongo drums) for **≤** 2 years between the ages of 8 and 15 years. Musicians had been trained to play either the violin (*N* = 15), flute (*N* = 16), or trumpet (*N* = 6) but had never been trained to play either of the other two instruments. Musicians had 3–53 years (median = 15 years, IQR = 7) of musical training, which they started at age 3–16 years (median = 7 years, IQR = 3). They had started playing the violin, flute, or trumpet at age 3–34 years (median = 8 years, IQR = 3) and had played the instrument for 2–53 years (median = 11 years, IQR = 12). Table I displays the demographics of musicians separated by training group (violinists, flautists, and trumpeters); independent-samples Kruskal–Wallis tests showed no significant differences in these demographics among training groups [*H*(37) ≤ 5.5, *p* ≥ 0.063]. An independent-samples Mann–Whitney U test showed no significant difference in age between musicians and non-musicians (*U* = 208.5, *p* = 0.29).

**TABLE I.** Demographics for the three musician groups and the non-musician group. The table displays medians (except in the final row, where percentages are displayed), with interquartile ranges in parentheses. Rows 2 and 3 indicate musical training on any instrument, whereas rows 4 and 5 indicate musical training on the instrument of interest (the violin, flute, or trumpet). For non-musicians, participants who had never had musical training (*N* = 9) were excluded from the “age started music training” calculations.

	Violinists	Flautists	Trumpeters	Non-musicians
Age	27.0 (12.8)	21.9 (5.8)	24.9 (6.4)	25.5 (9.5)
Years of music training	19.0 (7.5)	14.5 (4.3)	13.5 (9.3)	0.0 (0.8)
Age started music training	6.0 (4.0)	6.5 (2.3)	7.5 (3.3)	11.0 (6.0)
Years playing trained instrument	19.0 (10.0)	9.0 (9.0)	11.0 (7.3)	—
Age started playing trained instrument	7.0 (2.5)	10.0 (3.3)	10.0 (2.3)	—
Percentage currently practicing trained instrument (%)	73.3	43.8	66.7	0.0

The experiment was cleared by Western University's Health Sciences Research Ethics Board and the University College London (UCL) Research Ethics Committee. Informed consent was obtained from all participants. Seventeen participants (6 violinists, 7 flautists, and 4 trumpeters) took part at Western University, and 34 (9 violinists, 9 flautists, 2 trumpeters, and 14 non-musicians) participated at UCL.

### B. Apparatus

The experiment was conducted in a sound-attenuating booth. Participants sat in a comfortable chair facing an LCD visual display unit.

Acoustic stimuli were presented through an external sound card [Steinberg UR22 sound card (Steinberg Media Technologies) at Western University or ESI Maya 22 USB (ESI Audiotechnik GmbH) at UCL]. Stimuli were delivered binaurally through earphones [Etymotic Research ER1 earphones (Etymotic Research, Inc., Elk Grove Village, IL) at Western University], which were sealed in the ear canal of the participant with disposable foam inserts, or circumaural headphones [Sennheiser HD 380 Pro (Sennheiser Electronic GmbH & Co. KG, Wedemark, Germany) at UCL].

### C. Stimuli

We constructed 33 unique four-tone sequences using concert pitches between C4 (261.6 Hz) and C5 (523.3 Hz). The sequences included a range of patterns (tonal and triadic sequences, chromatic sequences, and atonal patterns) and a variety of intervals and melodic contours. Within each four-tone sequence, the musical pitch of every tone was different. Across sequences, there was approximately equal representation of each of the 13 pitches in the C4–C5 octave.

**FIG. 1.**
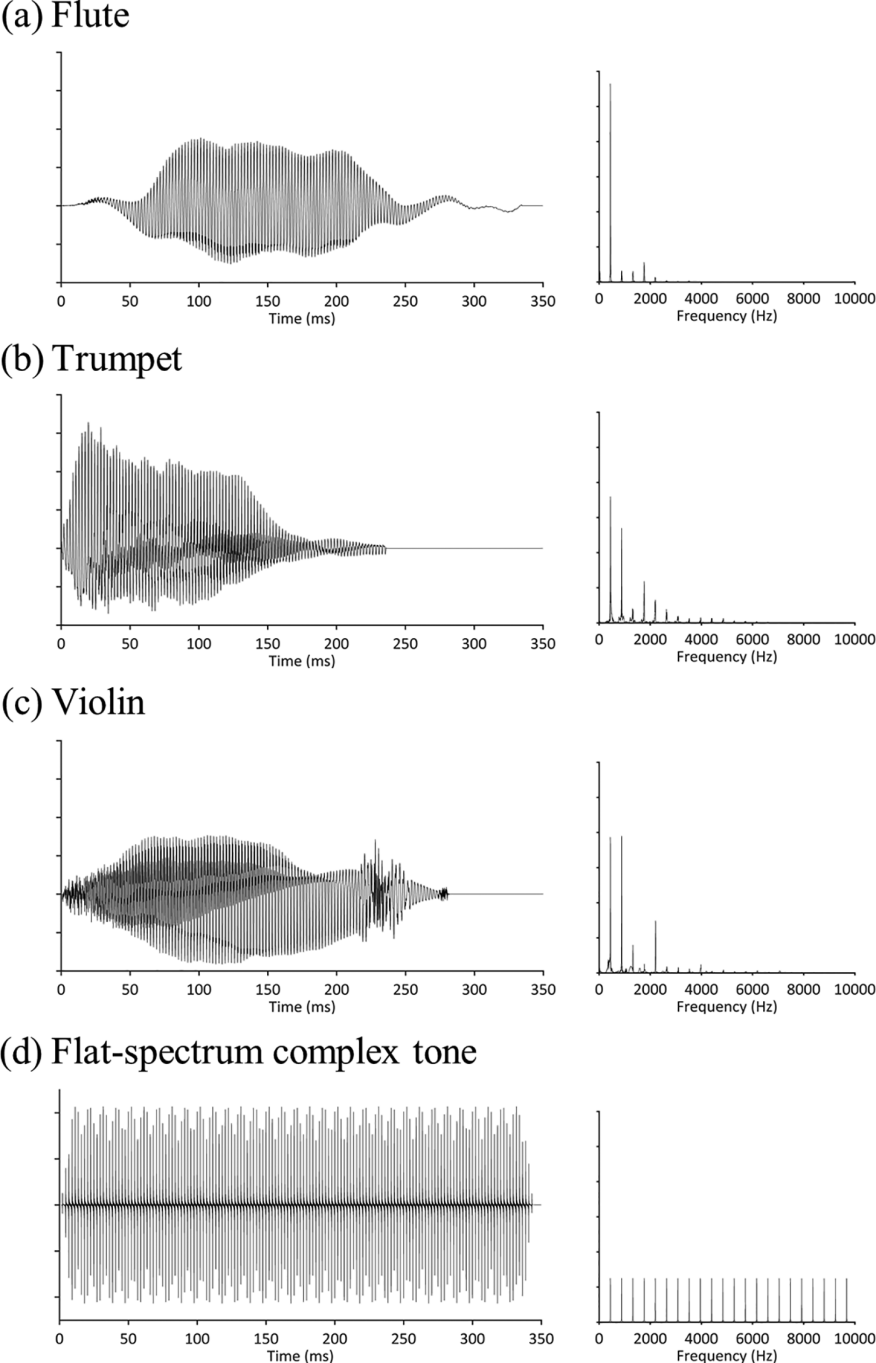
Waveforms (left) and spectra (right) of example stimuli at A4 pitch. (a) Flute; (b) trumpet; (c) violin; (d) flat-spectrum complex tone.

Violin, trumpet, and flute tones were obtained from the Philharmonia Orchestra sound samples library (https://philharmonia.co.uk/resources/sound-samples/) (for examples, see Fig. 1). We selected tones of approximately 350-ms duration from the samples. We also created artificial flat-spectrum harmonic complex tones of 350-ms duration using custom-written matlab scripts (version 2014 b; MathWorks, Inc., Natick, MA). These harmonic complex tones were created by summing sine waves (with 0° phase) at integer multiples of the fundamental frequency up to 20 000 Hz. A ramp time of 10 ms was applied to the beginning and the end of each tone. All stimuli were normalised to the same root mean square (rms) amplitude and were presented at a comfortable listening level (approximately 70–80 dB A), which was always well above threshold but differed across participants.

To create deviant-pitch tones, we shifted the pitch of individual tones upward (i.e., so that they sounded sharper than the original) using the “Change Gender” function in Praat (Boersma and Weenink, 2005) to specify a new median pitch. This procedure changed the frequencies of the harmonics within the tone at ratio intervals of 0.0005, producing tones with higher pitches than the originals. We chose to always shift the pitch upward because thresholds for detecting pitch increases and pitch decreases may differ (e.g., Salzberg, 1980).

**FIG. 2.**
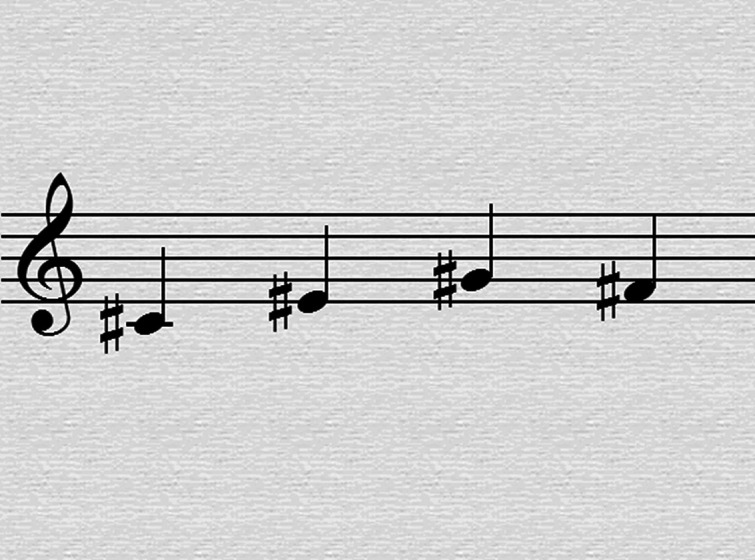
Musical notation for one of the 33 four-tone sequences, which was displayed to participants before they heard the sequence.

We showed participants musical notation for each four-tone sequence before the sequence began, so that they knew in advance the correct pitches of the tones. Each of the 33 four-tone sequences was illustrated as one bar of music in common (4/4) time (Fig. 2). Each tone was displayed as a quarter note. The stimuli were written in simple Western notation using Sibelius 8.0 (Avid Technology, Inc., Burlington, MA). All sequences were written in the treble clef, but with no time signature, key signature, dynamics, tempo, or other expressive markings indicated. Because the acoustic stimuli did not distinguish between enharmonic equivalents (e.g., F-sharp and G-flat, or B-natural and C-flat), the most common enharmonic equivalents were displayed with approximately equal frequency across sequences.

### D. Procedure

On each trial, the visual notation was presented on the screen for 1500 ms. Next, after an inter-stimulus interval of 700 ms, participants heard two repetitions of the four-tone sequence that had been displayed visually. Adjacent tones were presented with a stimulus onset asynchrony (SOA) of 350 ms. During the second presentation of each sequence, either the second or third tone of the sequence was higher in pitch than the equivalent tone in the first presentation. Participants had to respond which of the two tones was “mistuned” in a two-alternative forced-choice procedure. The tones within each trial were always the same timbre (violin, trumpet, flute, or artificial harmonic complex tones).

We measured each participant's 70.0% discrimination threshold using a weighted up-down adaptive procedure (Kaenbach, 1991; step size = 0.0005, weighting ratio of 3:7). The starting value for each run was 0.0102 above the median pitch of the original tone (e.g., 264.3 Hz for an original pitch of 261.6 Hz). The procedure stopped after eight reversals. We adapted pitch for each timbre (violin, flute, trumpet, artificial complex tone) in four separate, but interleaved, runs. Therefore, the timbre varied randomly from one trial to the next. JNDs were calculated as the median of the last five reversals for each run. We express the 70.0% JND as the Weber fraction, which is calculated as the absolute difference in pitch at the 70.0% threshold divided by the reference pitch.

### E. Analyses

Analyses were performed in SPSS, and effect sizes were calculated using MOTE (Buchanan *et al.*, 2019).

To examine effects of musicianship on pitch discrimination thresholds, we performed a two-way mixed analysis of variance (ANOVA), with timbre (violin, flute, trumpet, and complex tone) as a within-subjects factor and musicianship (musician and non-musician) as a between-subjects factor. For the musician group, we collapsed across flautists, violinists, and trumpeters.

To examine effects of instrument-specific training on pitch discrimination thresholds, we analysed the data from musicians across the three training groups (violinists, flautists, and trumpeters). We analysed the results using a two-way mixed ANOVA, with timbre as a within-subjects factor and training as a between-subjects factor. To examine differences between timbres—which were either natural musical instrument sounds or artificial flat-spectrum harmonic complex tones—we performed (planned) paired-samples *t*-tests to compare thresholds between the four timbres.

Given that we hypothesised there would be differences in discrimination thresholds depending on whether or not participants had learnt the instrument that produced the stimulus timbre, we performed three planned independent-samples *t*-tests: one compared thresholds for violin tones depending on whether or not participants had learnt to play the violin (i.e., violinists compared to flautists and trumpeters), and the other two tests compared thresholds for flute and trumpet tones depending on whether or not participants had learnt to play the flute or trumpet, respectively.

Given there was wide variability in musical history within each musician group, we examined the relationship between these general training factors and pitch discrimination thresholds. All musicians (*N* = 37) were included in these analyses. The training data violated the assumption of normality, so we used Spearman's rank correlation coefficients to examine these relationships. First, we calculated the difference in thresholds between the artificial flat-spectrum complex tones and the average of the three natural musical instrument timbres. We then examined the relationship between this threshold difference and (i) the number of years of musical experience for each participant and (ii) the age at which the participant began musical training. We also calculated the threshold benefit for the trained instrument in each participant, defined as the difference in thresholds between the trained instrument (e.g., violin tones for violinists) and the average of the thresholds for the two other instruments (e.g., flute and trumpet tones for violinists). We examined the relationship between the threshold benefit for the trained instrument and (i) the number of years participants had played that instrument (e.g., the number of years playing the violin for violinists) and (ii) the age at which participants had started learning that instrument. In addition, we compared the threshold benefit for the trained instrument between musicians who regularly played their trained instrument (who reported practicing every week, on average; *N* = 22) and those who were no longer practicing it (*N* = 15), using an independent-samples *t*-test.

## III. RESULTS

### A. Musicians compared to non-musicians

**FIG. 3.**
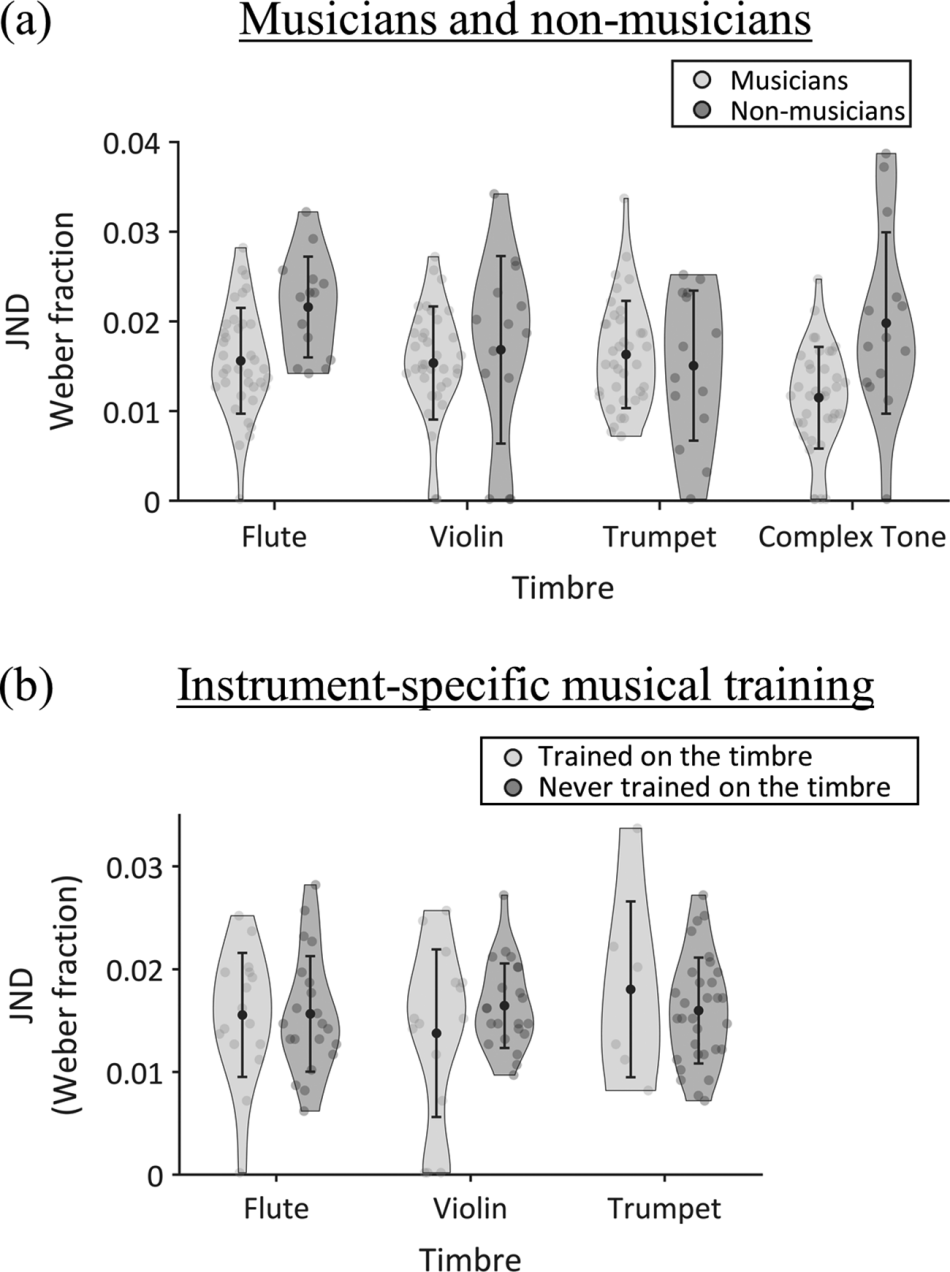
Results from the pitch discrimination task, displayed on violin plots. (a) JND for pitch discrimination of sounds of each timbre (flute tones, violin tones, trumpet tones, and flat-spectrum complex tones). Grey dots show results from individual participants, black dots show the mean for each group and timbre, and error bars display one s.d. of the mean. (b) JND for the three musical timbres (flute tones, violin tones, trumpet tones), separated by participants who were trained and those who were not trained to play the corresponding instrument (e.g., for flute tones, flautists compared with violinists and trumpeters).

Figure 3(a) illustrates thresholds across the four timbres for musicians and non-musicians. There was no significant main effect of timbre: *F*(3, 147) = 2.27, *p* = 0.08, *ω_p_*^2^ = 0.02 [95% confidence interval (CI): 0.00–0.07]. However, there was a significant main effect of musicianship [*F*(1, 49) = 7.02, *p* = 0.011, *ω_p_*^2^ = 0.11 (95% CI: 0.00–0.30)]: musicians [mean = 0.0147, standard deviation (s.d.) = 0.0044] had significantly smaller (i.e., better) thresholds than non-musicians (mean = 0.0183, s.d. = 0.0044).

There was also a significant interaction between timbre and musicianship [*F*(3, 147) = 5.39, *p* = 0.002, *ω_p_*^2^ = 0.07 (95% CI: 0.00–0.15)]. Independent-samples *t*-tests showed that musicians had smaller thresholds than non-musicians for the flat-spectrum harmonic complex tones [*t*(15.97)= 2.91, *p* = 0.010, *d* = 0.83 (95% CI: 0.27–1.55)] and flute tones [*t*(49) = 3.23, *p* = 0.002, *d* = 0.92 (95% CI: 0.36–1.66)] but not for the violin tones [*t*(16.72) = 0.50, *p* = 0.62, *d* = 0.14 (95% CI: –0.46–0.77)] or trumpet tones [*t*(49) = 0.67, *p* = 0.50, *d* = 0.19 (95% CI: –0.41–0.83)]. Looking at the interaction the other way using one-way repeated measures ANOVAs for each group, there was a significant effect of timbre for musicians [*F*(3, 108) = 7.80, *p* < 0.001, *ω*^2^ = 0.36 (95% CI: 0.20–0.48)] but not for non-musicians [*F*(1.87, 24.4) = 1.72, *p* = 0.20, *ω*^2^ = 0.09 (95% CI: 0.00–0.33)]. The significant effect of timbre in musicians was underpinned by smaller thresholds for flat-spectrum harmonic complex tones than for the three musical instrument timbres [violin: *t*(36) = 3.88, *p* < 0.001, *d* = 0.64 (95% CI: 0.28–0.99); flute: *t*(36) = 3.73, *p* = 0.001, *d* = 0.61 (95% CI: 0.26–0.96); trumpet: *t*(36) = 5.12, *p* < 0.001, *d* = 0.84 (95% CI: 0.46–1.21)]; there were no significant differences among pairs of the three musical instrument timbres [*t*(36) ≤ 0.92, *p* ≥ 0.37, *d* ≤ 0.15].

### B. Instrument-specific musical training

Table II separates thresholds for each of the timbres (violin, flute, trumpet, and flat-spectrum harmonic complex tone) by instrument-specific training group (violinists, flautists, and trumpeters).

**TABLE II.** Mean JNDs (Weber fractions) for each instrument-specific training group across the four timbres. Parentheses indicate one s.d. of the mean.

	Timbre			
Training group	Violin	Flute	Trumpet	Flat-spectrum complex tone
Violinists	0.014 (0.008)	0.014 (0.005)	0.014 (0.006)	0.013 (0.006)
Flautists	0.016 (0.003)	0.016 (0.006)	0.018 (0.004)	0.011 (0.004)
Trumpeters	0.019 (0.005)	0.019 (0.005)	0.018 (0.009)	0.007 (0.005)

There was a significant main effect of timbre [*F*(3, 102)= 8.68, *p* < 0.001, *ω_p_*^2^ = 0.17 (95% CI: 0.04–0.29)]. Consistent with the analyses reported in Sec. III A, planned contrasts revealed that listeners had smaller thresholds for flat-spectrum harmonic complex tones than for violin [*t*(36)= 2.83, *p* = 0.008, *d_z_* = 0.47 (95% CI: 0.12–0.80)], flute [*t*(36) = 3.07, *p* = 0.004, *d_z_* = 0.50 (95% CI: 0.16–0.84)], and trumpet [*t*(36) = 3.47, *p* = 0.001, *d_z_* = 0.57 (95% CI: 0.22–0.92)] tones. Thresholds did not differ significantly between the violin, flute, and trumpet tones [*t*(36) ≤ 0.78, *p* ≥ 0.44, *d_z_* ≤ 0.13].

The main effect of instrument-specific training was not significant [*F*(2, 34) = 0.89, *p* = 0.42, *ω_p_*^2^ = –0.01 (95% CI: 0.00–1.00)], showing that there were no overall differences in discrimination thresholds between participants who were trained on different instruments. There was a significant interaction between timbre and instrument-specific training [*F*(6, 102)= 2.34, *p* = 0.037, *ω_p_*^2^ = 0.06 (95% CI: 0.00–0.12)], showing that thresholds for each timbre differed depending on the instrument that participants were trained to play. However, the interaction was not underpinned by significant differences between training groups for any of the four timbres: between-subjects one-way ANOVAs at each timbre showed no significant effects of instrument-specific training [violin tones: *F*(2, 36) = 1.40, *p* = 0.26, *ω*^2^ = 0.02 (95% CI: 0.00–0.15); flute tones: *F*(2, 36) = 1.58, *p* = 0.22, *ω*^2^ = 0.03 (95% CI: 0.00–0.18); trumpet tones: *F*(2, 36) = 1.75, *p* = 0.19, *ω*^2^ = 0.04 (95% CI: 0.00–0.20); flat-spectrum complex tones: *F*(2, 36) = 3.21, *p* = 0.053, *ω*^2^ = –0.11 (95% CI: 0.00–0.31)]. Instead, the interaction was underpinned by significantly smaller thresholds for the flat-spectrum harmonic complex tone than other timbres among flautists [*F*(3, 45) = 5.08, *p* = 0.004, *ω*^2^ = 0.19 (95% CI: 0.00–0.38)] and trumpeters [*F*(3, 15) = 5.32, *p* = 0.011, *ω*^2^ = 0.39 (95% CI: 0.00–0.64)], whereas the difference was not significant for violinists [*F*(3, 42) = 0.08, *p* = 0.97, *ω*^2^ = –0.06 (95% CI: 0.00–1.00)].

**FIG. 4.**
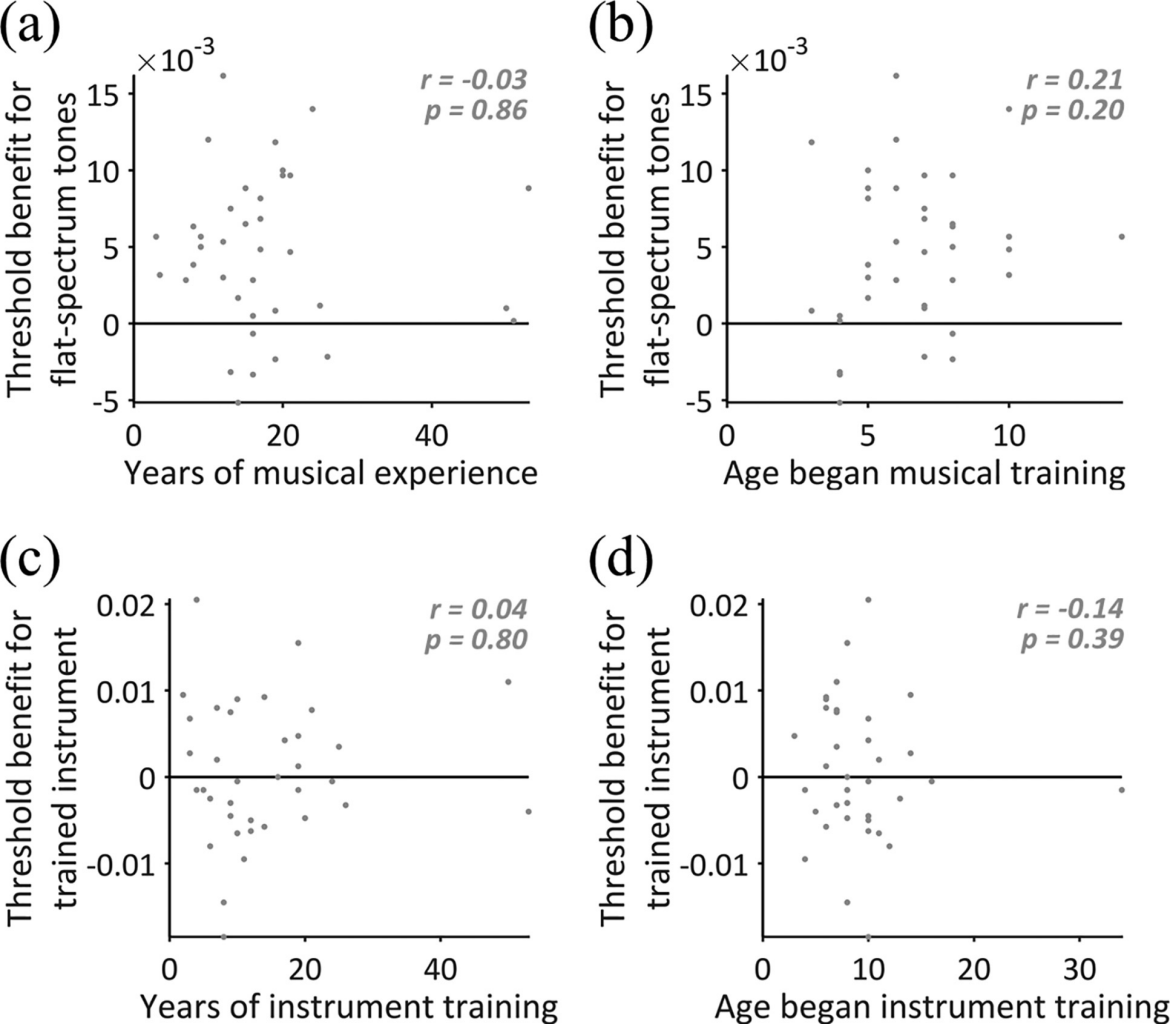
Scatter plots showing relationships between training variables and thresholds. The upper row shows relationships between the threshold benefit for flat-spectrum complex tones compared to musical tones and (a) the number of years participants received musical training and (b) the age participants had begun musical training. The lower row shows relationships between the threshold benefit for the trained instrument compared to untrained instruments and (c) the number of years participants had been trained on the instrument and (d) the age participants had started learning the trained instrument. Each dot shows an individual participant (musicians only).

Given that there was a trend for violinists to have the most musical training (see Table I), we investigated whether musical training variables influenced the threshold benefit for the flat-spectrum complex tone (i.e., difference in JND between the flat-spectrum complex tone and the average of the three musical instrument timbres). The magnitude of the threshold benefit did not correlate with the number of years of musical training [*r* = –0.03, *p* = 0.86; Fig. 4(a)] or the age at which the participant began musical training [*r* = 0.21, *p* = 0.20; Fig. 4(b)].

Figure 3(b) separates thresholds for each of the three instruments by whether participants were trained on the timbre or not. Planned independent-samples *t*-tests confirmed there was no significant difference in thresholds for violin tones depending on whether or not participants were trained to play the violin [*t*(18.79) = 1.14, *p* = 0.27, *d* = 0.39 (95% CI: –0.28–1.04)], no significant difference in thresholds for flute tones depending on whether or not participants were trained to play the flute [*t*(35) = 0.05, *p* = 0.96, *d* = 0.02 (95% CI: –0.63–0.67)], and no significant difference in thresholds for trumpet tones depending on whether or not participants were trained to play the trumpet [*t*(5.62) = 0.52, *p* = 0.62, *d* = 0.18 (95% CI: –0.65–1.11)].

To investigate whether musical training variables influenced the threshold benefit for the trained instrument (i.e., difference in JND between the trained and the average of two untrained instruments), we calculated Spearman's rank correlation coefficients. The magnitude of the threshold benefit did not correlate with the number of years of training on the instrument [*r* = 0.04, *p* = 0.80; Fig. 4(c)] or the age at which the participant began learning the trained instrument [*r* = –0.14, *p* = 0.39; Fig. 4(d)]. There was no difference in the threshold benefit between participants who regularly played their trained instrument (mean = 0.0025, s.d. = 0.0076) and those who were no longer practicing it at the time of the experiment (mean = –0.0017, s.d. = 0.0082) [*t*(35) = 1.57, *p* = 0.13, *d* = 0.53 (95% CI: –0.15–1.19)].

## IV. DISCUSSION

We found that flautists and trumpeters had better thresholds for discriminating the pitch of flat-spectrum harmonic complex tones than of natural violin, flute, or trumpet tones. The significant interaction we found between instrument-specific training and timbre reflects a greater contribution of acoustics to pitch discrimination than of familiarity with sounds of natural timbres. Our finding implies that flautists and trumpeters can use the acoustic content of flat-spectrum harmonic complex tones (e.g., spectral content, jitter, harmonic-to-noise ratio) to help discriminate pitch. In contrast, a benefit based on familiarity would lead to better thresholds for notes played by musical instruments—because those sounds are heard regularly in everyday life—than for artificial flat-spectrum tones. In addition, we found that thresholds were no better for sounds produced by the instrument that a musician is trained to play than by instruments that they have never been trained to play. This finding suggests that extensive experience with (playing and listening to sounds of) one instrument does not improve pitch discrimination of sounds from that instrument over discrimination of sounds from other musical instruments belonging to different instrument families (i.e., wind, brass, and string).

Our finding that pitch discrimination thresholds do not differ reliably between trained and untrained instruments suggests that memory representations of, and sensorimotor mappings for, pitches of trained instruments do not reliably help listeners to discriminate smaller differences in pitch for timbres of trained instruments than less familiar timbres. We also found no relationship between discrimination benefits for trained compared to untrained instruments and either the age that participants started learning the trained instrument or the number of years they played it, suggesting that even extensive experience with sounds of particular timbres does not help discriminate pitch for those timbres over other, untrained, timbres. Possibly, familiarity with the envelopes or spectra of known timbres and/or enhanced neural representations for sounds played by familiar instruments (Pantev *et al.*, 2001; Shahin *et al.*, 2008; Strait *et al.*, 2012) help musicians to perform other tasks, but our findings imply that they are not used to improve pitch discrimination.

Surprisingly, we found that flautists and trumpeters had better discrimination thresholds for artificial complex tones than for musical instrument tones, whereas violinists showed no difference among timbres—and the difference among instrument-specific training groups (i.e., between flautists, trumpeters, and violinists) was confirmed by a significant interaction. Nevertheless, violinists showed a trend in the same direction. There were no significant differences in training demographics among the groups, although there was a trend for the violinists that we recruited to have started training younger and to have continued training for longer than the flautists and trumpeters. Thus, one possible explanation is that earlier and longer musical experience helps to override acoustic advantages for pitch discrimination of flat-spectrum harmonic complex tones. However, we found no significant correlation between the threshold benefit for artificial complex tones (compared with natural instrument tones) and either the age at which participants began musical training or the number of years they received musical training—which is inconsistent with this explanation. Possibly, given that violinists had smaller (albeit not significantly smaller) thresholds for all timbres than flautists and trumpeters, our task may have been less able to detect differences among timbres in violinists. Conversely, this result may reflect true differences in thresholds among musicians who play different instruments, which have been previously reported (e.g., Micheyl *et al.*, 2006), albeit not for the combination of musician groups (violinists, flautists, and trumpeters) studied here. For example, violinists need to tune their instruments and make online adjustments to pitch based on their finger position, whereas flautists and trumpeters do not tune their instruments and make online adjustments to pitch based on their mouth position. Possibly, this difference could confer a small advantage for discriminating the pitch of natural instrument sounds in violinists, thereby reducing the difference in pitch discrimination between natural and artificial tones.

Consistent with the results of previous studies (e.g., Besson *et al.*, 2007; Brown *et al.*, 2017; Kishon-Rabin *et al.*, 2001; Magne *et al.*, 2006; Micheyl *et al.*, 2006; Spiegel and Watson, 1984), we found better pitch discrimination overall in musicians than non-musicians, suggesting that—in general—musical training improves pitch discrimination. We found that the difference between musicians and non-musicians is most pronounced for artificial flat-spectrum tones and flute tones. A previous study by Micheyl *et al.* (2006) showed a greater musician benefit for artificial harmonic complex tones than for pure tones. Our results extend those of Micheyl *et al.* (2006) by showing that the greater musician benefit for artificial complex tones holds when compared to natural complex tones, such as those produced by the flute. In the current study, participants were presented with musical notation at the beginning of each trial, which could have been more useful for musicians than for non-musicians. However, differences in the use of musical notation do not seem to have affected our results: We found no significant difference between musicians and non-musicians for violin tones and trumpet tones, so it is unlikely that musical notation contributed to differences between groups for the other timbres.

Our results suggest that musical experience (specifically, training on the flute and trumpet) allows participants to better utilise the spectral content of flat-spectrum harmonic complex tones (e.g., more harmonics or an equal distribution of intensity across harmonics) to help discriminate the pitches of these sounds, compared to natural sounds with a skewed distribution of harmonic intensities. Given the results of Moore *et al.* (2019), this effect is likely due to better auditory sensitivity to pitch cues in musicians rather than sharper frequency selectivity. However, the mechanism underlying the advantage for flat-spectrum harmonic complex tones is unclear. At the lower harmonics, the distribution of harmonic intensities is equal for artificial flat-spectrum tones, whereas it is skewed for natural tones—and musicians may be able to use the equal harmonic intensities to discriminate smaller differences in pitch. Alternatively, they may be able to better utilise information at higher harmonics that are present in artificial flat-spectrum tones and absent in natural tones. While there is some evidence that the higher harmonics contribute little to pitch perception among musicians (Dai, 2000; Moore *et al.*, 1985; Plack and Oxenham, 2005), Bernstein and Oxenham (2003) found that pitch discrimination for complex tones that contained only the ninth and higher harmonics was just as robust as for tones that contained lower harmonics. Therefore, musical training could allow musicians to better make use of the higher harmonics—for example, by taking advantage of the information contained at higher resolved harmonics or perhaps even allowing them to make use of temporal cues conferred by the beating of harmonics within unresolved high-frequency channels (Grimault *et al.*, 2002). Dai (2010) suggests that unresolved harmonics do not affect pitch perception in non-musicians, although whether musicians utilise this information is unknown. Another possibility is that the flat amplitude envelope of the artificial complex tones we used (see Fig. 1) may enable musicians to more reliably extract pitch information. However, given natural music sounds typically have modulated amplitude envelopes and contain none of the higher harmonics (see Fig. 1), this advantage cannot be due to direct experience with sounds. The flat-spectrum harmonic complex tones we used may evoke a more salient pitch than the natural instrument sounds; an analysis of our stimuli shows that the artificial tones have less jitter and a greater harmonic-to-noise ratio than the natural instrument tones (Table III). However, our finding that discrimination thresholds in musicians are best for flat-spectrum tones is unlikely to be due to salience conferred by acoustics, because under this explanation, we should have found better thresholds for flat-spectrum complex tones in non-musicians and in all of the sub-groups of musicians, although perhaps musicians are better at extracting pitch from natural sounds despite jitter and a lower harmonic-to-noise ratio. Another possibility is that the advantage relates to better working memory for frequency in people with musical experience, which has been previously demonstrated for pure tones (Lad *et al.*, 2020; Lad *et al.*, 2022). Our task likely engaged working memory, because it required participants to compare tones that were separated by 1050 ms. Nevertheless, it is unclear why a working memory advantage would be evident for flat-spectrum harmonic complex tones and not for musical instrument tones. Overall, our results suggest that musical training can help listeners to better extract, or hold in memory, pitch based on the spectral content of flat-spectrum harmonic complex tones relative to complex tones with a skewed distribution of harmonic intensities—reflecting a musician advantage that has not previously been reported.

**TABLE III.** Summary of the mean acoustic properties for the tones of each timbre. Parentheses indicate one s.d. of the mean. Jitter refers to the average absolute difference between consecutive periods, divided by the average period. The strength of the autocorrelation is the global peak value from the normalised autocorrelation function. The harmonic-to-noise ratio is the mean across the stimulus.

Acoustic property	Violin	Flute	Trumpet	Flat-spectrum complex tone
Jitter	0.0051 (0.0051)	0.0055 (0.0023)	0.0039 (0.0015)	<0.0001 (<0.0001)
Strength of autocorrelation	1.00 (0.00)	1.00 (0.00)	1.00 (0.00)	1.00 (0.00)
Harmonic-to-noise ratio	23.4 (3.0)	22.6 (4.1)	21.5 (2.3)	52.5 (7.4)

Broadly speaking, our results add to a wide literature showing that pitch interacts with timbre, demonstrating that these two dimensions are not perceived independently. For example, previous studies have shown that a change in timbre affects pitch comparisons (e.g., Allen and Oxenham, 2014; Krumhansl and Iverson, 1992; Melara and Marks, 1990a, 1990b; Pitt, 1994; Singh and Hirsh, 1992; Vurma *et al.*, 2011; Warrier and Zatorre, 2002). Functional imaging data and research on neurological patients have revealed partially separate neural substrates for pitch and timbre—implicating timbre processing at a higher level—although with some overlap at early stages of cortical processing (Griffiths *et al.*, 2007; Warren *et al.*, 2005), consistent with behavioral interactions between pitch and timbre. Our study expands upon previous behavioral findings by showing that—even when timbre is held constant—pitch discrimination depends on acoustics of timbre. Specifically, for musicians, we demonstrated better pitch discrimination for artificial complex tones that contain equal amplitude harmonics than for natural complex tones with a skewed distribution of harmonic intensities, despite greater familiarity with natural sounds.

The pitch discrimination thresholds that we obtained for complex tones in musicians (Weber fraction for 70.0% JND = 0.015) are higher than those reported for discrimination of complex tones by musicians in three previous studies [Allen and Oxenham (2014): Weber fraction for 70.7% JND of approximately 0.8%, corresponding to a Weber fraction of 0.008; Brown *et al.* (2017): 70.7% JND of approximately 8 cents, corresponding to a Weber fraction of 0.005; Micheyl *et al.* (2006): Weber fraction for 70.7% JND = 0.001]. However, those previous experiments presented two complex tones sequentially with a brief inter-stimulus interval, rather than embedded within a sequence of tones. Given that Spiegel and Watson (1984) found that musicians had higher pitch discrimination thresholds when they discriminated between pure tones within ten-tone sequences (Weber fraction = 0.06) than between pure tones presented sequentially (Weber fraction = 0.001–0.005), higher thresholds for complex tones in the current experiment than in previous experiments presenting two tones sequentially (Brown *et al.*, 2017: Micheyl *et al.*, 2006) could occur because our complex tones were embedded within four-tone sequences. Another possible explanation for differences in thresholds between experiments is tone duration, which is known to influence discrimination thresholds (see Gockel *et al.*, 2007; Kidd and Watson, 1992). The alternative explanation that thresholds in the current experiment were higher than in previous experiments because the musicians did not have sufficient musical training is unlikely because Besson *et al.* (2007) found improvements in pitch discrimination thresholds after only 6 months of musical training—and all of our musicians had musical training for 3 years or longer.

We do not have information about whether our participants were able to use absolute pitch, but we do not expect this to influence the results. For example, Micheyl *et al.* (2006) found no difference in pitch discrimination for pure and complex tones between musicians who possessed absolute pitch and those who did not.

In conclusion, we found that musical experience plays a role in enhancing pitch discrimination abilities—and this is due to a better ability to make use of the spectral content of (artificial) stimuli with flat spectra rather than an enhancement for timbres most similar to those that have been trained (i.e., natural musical instrument sounds). We predicted that natural familiarity with an instrument would lead to better thresholds for discriminating pitch, but we found no evidence for an advantage. Pitch discrimination thresholds were no better for sounds with highly familiar timbres (i.e., those of instruments the participants were trained to play) than for sounds with less familiar timbres. In fact, pitch discrimination thresholds were best (and the musician advantage was the greatest) for the most unfamiliar type of sound—a flat-spectrum harmonic complex tone—indicating that acoustics influence pitch discrimination thresholds more than does timbral familiarity.
